# A Case of Hæmorrhage of the Alveola, with Remarks upon Odontome, Osteome and Osteo-Odontome

**Published:** 1869-12

**Authors:** O. Salomon

**Affiliations:** (Berlin).


### ARTICLE III.

### *A Case of Hæmorrhage of the Alveola, with Remarks upon Odontome, Osteome and Osteo-odontome.*

By Dr. O. Salomon, (Berlin).

In the monthly Association of the Physicians in Heiemark Dr. Tanzer, Doient of Dental Surgery in the University of Graz, related the following cases, which I think ought to be mentioned.

The first case is that of Mr. G. T., officer in the Austrian army, aged 64. He was of robust and plethoric constitution. His previous health had been excellent, and he had never suffered from hæmorrhage after the extraction of teeth or incised wounds. On the 9th of September, 1868, he went to the office of Dr. Tanzer, suffering from a severe periostitis alveolaris, and periodontitis of the second right lower molar. Chloroform, tannin, carbolic acid, the local application of three leeches, and morphia internally had no effect whatever. On the third day, at 4 o'clock in the afternoon, the tooth was so sensitive that the slightest touch caused the greatest pain, and it became necessary to remove it with local anæsthesia. The hæmorrhage was an ordinary one and stopped completely after twenty minutes. Early on the following day the patient returned again, and stated that the hæmorrhage had commenced at 11 o'clock the previous evening and notwithstanding the application, by a surgeon, of the liquor ferri, it continued profusely. Dr. Tanzer applied the liquor ferri sesqui-chloridi, with ice water internally and and the mouth gargled with alum and tannin, used a tampon, a plaster of paris impression and afterward a gutta-percha impression, fastened by means of a bandage around the jaw and temples, but all to no purpose. Finally, it being impossible to replant the extracted tooth, owing to the great sensibility of the alveola, and not liking to use the actual cautery, Dr. T. concluded to apply a compress, which was made from a silver plate, and provided with a gold clasp to fasten on the adjacent teeth.*

At 7 P. M., the same day, this compressor was applied over a tampon saturated with carbolic acid. Immediately the hæmorrhage ceased. Every two days the compress was removed to be reapplied, after cleansing the mouth. During these removals there twice appeared a small hæmorrhage. The patient continued to use this apparatus until the cavity in the alveolar was filled with granulations. The literature of the subject shows a great many cases of alveolar hæmorrhage, resulting from the extraction of teeth and terminating in death. In many it was necessary to employ the actual cautery, in many to tie the carotids. I am perfectly convinced that a resort to these agents is unnecessary, and that the compress will answer in all cases. I am not very fond of these instruments of torture from the middle ages, such as caustics, vesicants, blood letting, etc., and I have always avoided them in my practice. I am glad to learn the opinion of Dr. Niemeyer upon a case of alveolar hæmorrhage, resulting from the extraction of a tooth.§ He declares that after the failure of the actual cautery (twice applied) and all the therapeutical agents which it was possible to employ the patient's life might have been saved, had there been present a dentist to take the impression of the adjoining teeth, and adapt an artificial compressor to them. (It is to be regretted that in diseases of the gums and mouth, physicians do not seek consultation with dentists). Dissection of the tooth in the case of Dr. T. showed in the body odontome and in the roots osteome, both new formations. In the central portion, the canal was free, both full of old pulp follicles. The root of the pulp was obliterated, which in Dr. T 's opinion was the cause of the severe periostitis, alveolaris and periodontitis, and the inefficacy of all therapeutical measures.

* Why not gold alone, or, if too expensive, vulcanite rubber. The combination of two metals will cause an electrical action in the mouth.—Dr. O. S.

§ American Journal of Dental Science, June, 1869. Death from Hemorrhage by Dr. O. Salomon.

The second case is that of Mr. S. H., aged 34, a merchant in Maehren. The patient had suffered for eight or nine years with a continuous pain of the last tooth of the lower jaw, sometimes on the right, sometimes on the left side. It was his custom, when he became tired of the pain, to have the painful tooth extracted, but at the end of four weeks the pain invariably returned in the next tooth to the one which had been extracted. In July, 1868, he came to Dr. T.'s office asking to have the second lower right bicuspid extracted. The tooth appeared perfectly healthy and he tried before extraction, all therapeutic remedies, without the least effect. He made an examination of all the extracted teeth, which the patient had preserved, and found the most beautiful example of pear-shaped odontome and osteo-odontome. After this examination he was compelled to extract the tooth, the pulp cavity of which was perfectly full of osteo-odontome and completely obliterated. Three weeks afterwards he prepared a partial set of artificial teeth for this patient. He arranged the teeth two lines higher than the tops of the other seven remaining teeth of the lower jaw, but a month after the patient informed him that the pain had begun again in the next tooth. In September this tooth was extracted and found filled with the same peculiar formation. In October again, the symptoms of pain showed themselves in the next tooth, the canine. The patient is now under the treatment of a specialist in Vienna.

This affection, in my opinion can only terminate on the removal of all the teeth. But the cause for the recurrence of the pain in the tooth next to the one removed is doubtful. It is probable that the new formation began in each tooth a long time previous to the extraction of the one behind it; possibly at the same time with the painful one.

Atrophia and perfect obliteration of the pulp canal occur very often in old age, but in persons between 24 and 36 years very seldom occur new formations of this kind. The best histological works and examination have been published by Tomes, Ulrich Wedl and Heider. In 1868 Prof. Dr. Hahl, of Halle wrote a scientific monographia.

He divided the new formation into

(1.) Odontome, Dentine, new formations.

(2.) Osteome, Cement, new formations.

(3.) Osteo-odontome, Dentine, and Cement, formations mixed.

### *East Tennessee Dental Association.*

